# Clinical and Economic Evaluation of a Real-Time Chest X-Ray Computer-Aided Detection System for Misplaced Endotracheal and Nasogastric Tubes and Pneumothorax in Emergency and Critical Care Settings: Protocol for a Cluster Randomized Controlled Trial

**DOI:** 10.2196/72928

**Published:** 2025-08-20

**Authors:** Chu-Lin Tsai, Teresa Cheng-Chieh Chu, Chih-Hung Wang, Wei-Tien Chang, Min-Shan Tsai, Shih-Chi Ku, Yen-Hung Lin, Hao-Chih Tai, Shuenn-Wen Kuo, Kuo-Chuan Wang, Anne Chao, Sung-Chun Tang, Wei-Lun Liu, Ming-Han Tsai, Ting-Ann Wang, Shu-Lin Chuang, Yi-Chia Lee, Lu-Cheng Kuo, Chiuan-Jung Chen, Jia-Horng Kao, Weichung Wang, Chien-Hua Huang

**Affiliations:** 1 Integrative Medical Data Center Department of Medical Research National Taiwan University Hospital Taipei Taiwan; 2 Department of Emergency Medicine National Taiwan University Hospital Taipei Taiwan; 3 Department of Internal Medicine National Taiwan University Hospital Taipei Taiwan; 4 Department of Surgery National Taiwan University Hospital Taipei Taiwan; 5 Department of Anesthesiology National Taiwan University Hospital Taipei Taiwan; 6 Stroke Center and Department of Neurology National Taiwan University Hospital Taipei Taiwan; 7 Division of Critical Care Medicine Department of Emergency and Critical Care Medicine Fu Jen Catholic University Hopsital New Taipei Taiwan; 8 Department of Critical Care Medicine Min-Sheng General Hospital Taoyuan Taiwan; 9 Center of Intelligent Healthcare National Taiwan University Hospital Taipei Taiwan; 10 Information Technology Office National Taiwan University Hospital Taipei Taiwan; 11 Hepatitis Research Center National Taiwan University Hospital Taipei Taiwan; 12 Institute of Applied Mathematical Sciences National Taiwan University Taipei Taiwan

**Keywords:** computer-aided detection system, artificial intelligence, pneumothorax diagnosis, endotracheal tube, nasogastric tube, clinical effectiveness, cost-effectiveness

## Abstract

**Background:**

Advancements in artificial intelligence (AI) have driven substantial breakthroughs in computer-aided detection (CAD) for chest x-ray (CXR) imaging. The National Taiwan University Hospital research team previously developed an AI-based emergency CXR system (Capstone project), which led to the creation of a CXR module. This CXR module has an established model supported by extensive research and is ready for application in clinical trials without requiring additional model training. This study will use 3 submodules of the system: detection of misplaced endotracheal tubes, detection of misplaced nasogastric tubes, and identification of pneumothorax.

**Objective:**

This study aims to apply a real-time CXR CAD system in emergency and critical care settings to evaluate its clinical and economic benefits without requiring additional CXR examinations or altering standard care and procedures. The study will evaluate the impact of CAD system on mortality reduction, postintubation complications, hospital stay duration, workload, and interpretation time, as wells as conduct a cost-effectiveness comparison with standard care.

**Methods:**

This study adopts a pilot trial and cluster randomized controlled trial design, with random assignment conducted at the ward level. In the intervention group, units are granted access to AI diagnostic results, while the control group continues standard care practices. Consent will be obtained from attending physicians, residents, and advanced practice nurses in each participating ward. Once consent is secured, these health care providers in the intervention group will be authorized to use the CAD system. Intervention units will have access to AI-generated interpretations, whereas control units will maintain routine medical procedures without access to the AI diagnostic outputs.

**Results:**

The study was funded in September 2024. Data collection is expected to last from January 2026 to December 2027.

**Conclusions:**

This study anticipates that the real-time CXR CAD system will automate the identification and detection of misplaced endotracheal and nasogastric tubes on CXRs, as well as assist clinicians in diagnosing pneumothorax. By reducing the workload of physicians, the system is expected to shorten the time required to detect tube misplacement and pneumothorax, decrease patient mortality and hospital stays, and ultimately lower health care costs.

**International Registered Report Identifier (IRRID):**

PRR1-10.2196/72928

## Introduction

### Background

Misplacement of medical tubes, such as endotracheal tube (ETT) and nasogastric tube (NGT), is a well-recognized clinical issue associated with serious complications, including pneumothorax, aspiration pneumonia, prolonged hospital stays, and even mortality [1-5]. Delayed management of pneumothorax could lead to tension pneumothorax, which is a life-threatening condition. In addition, misplacement of an ETT would cause hypoxia in critically ill patients. Misplacement of the NGT could lead to aspiration pneumonia. Taken together, these 3 conditions increase patient mortality, particularly related to respiratory causes. Portable chest radiography (chest x-ray [CXR]) is routinely performed in intensive care and emergency settings to confirm tube placement and detect complications. However, interpretation of portable CXR images can be challenging due to factors such as patient immobility and lower image quality, which may delay clinical decisions and increase the risk of adverse events. Therefore, with the rise of deep learning applications in the field of medical imaging, the development of artificial intelligence (AI) algorithms for CXRs has significantly enhanced the capabilities of image quality identification [6], and driving the development of computer-aided detection (CAD) systems for CXR imaging [7,8].

These systems support clinicians in diagnosing cardiopulmonary diseases, detecting pneumothorax, and preventing adverse events caused by improper tube placements. CXR imaging plays a vital role in assisting physicians with the diagnosis of cardiopulmonary conditions or pneumothorax and in identifying tube misplacements, as it is generally recommended to perform a CXR examination following the placement of ETT or NGT to confirm correct positioning [9,10]. Previous studies have demonstrated that deep learning models exhibit excellent performance in interpreting CXRs, particularly in detecting malpositioned tubes and thoracic abnormalities [11-15]. However, the development of these AI-CAD systems has typically relied on retrospective data and focused primarily on comparing their diagnostic accuracy with that of human clinicians. As a result, evidence remains limited regarding whether these AI-CAD systems can indeed deliver tangible clinical benefits when implemented in real-world clinical practice. Further validation through prospective studies, multicenter evaluations, and cost-effectiveness analyses is warranted to establish their real-world utility. Moreover, considering the increasing emphasis on health care sustainability and the need to allocate limited resources effectively, it is crucial to evaluate whether the adoption of AI-based CAD systems represents a worthwhile investment for health care systems.

In previous studies, we developed a real-time CAD system for emergency and critical care CXR imaging, incorporating deep learning technologies to detect mispositioned ETT and NGT as well as pneumothorax [16-18]. The system’s modules have demonstrated its potential to improve diagnostic efficiency, reduce adverse events related to tube misplacement, and enhance patient outcomes. However, its clinical efficacy and cost-effectiveness have yet to be validated through rigorous randomized clinical trial and economic evaluations. To bridge this gap, this project aims to conduct comprehensive clinical trials (prospective studies and multicenter evaluations) and cost-effectiveness assessments, thereby enhancing the system’s credibility and value for real-world clinical applications.

### Objective

This study aims to apply the AI-based CAD system for automatic detection and assessment of catheter placement and pneumothorax in emergency and intensive care settings. We aim to evaluate the clinical and economic benefits of the CAD system via rigorous randomized trial without altering routine care and procedures.

In terms of clinical benefits, the study will assess whether the use of the CAD system reduces mortality and will evaluate hospital stay duration, the time required to detect misplacement, and the time needed to reposition ETTs, NGTs, and chest drainage tubes. For the economic evaluation, the study will compare the cost-effectiveness of using the CAD system versus standard care without CAD.

### Prior Work

#### Overview

Our team selected a CXR module developed through the Capstone project (MOST 111-2634-F-002-015-, Capstone project) in collaboration with the commercial product provided by EBM Technologies, which has been approved by the ethics committee of National Taiwan University Hospital (NTUH; reference number: 202003106RINC). The CAD system automatically retrieves CXR images from the hospital’s Picture Archiving and Communication System, processes them within approximately 1 minute, and generates predictive results displayed on a centralized platform for clinical review. Alerts for abnormal findings are sent via text notifications to facilitate timely intervention. This system has been deployed in emergency and intensive care units (ICUs) to automate the identification and detection of mispositioned ETT and NGT on CXR images.

#### The Model of Localization and Detection of Malpositioned ETT

This model uses CXR images collected from NTUH, including both the Taipei main hospital and the Yunlin branch, which were divided into a training dataset and a testing dataset. The training dataset primarily consists of 5767 images collected from NTUH between 2015 and 2019. The testing dataset includes images from both chronological and geographical contexts: 955 images from NTUH during 2020 (NTUH-20 dataset) and 656 images from the NTUH Yunlin Branch (NTUH-YB dataset), representing different temporal and geographical backgrounds. During the image annotation process, the research team marked the position of the ETT and identified the instances of misplacement. The marking was done by nurse practitioners, emergency physicians, and radiologists with extensive clinical experience, and the images were classified according to whether the ETT was misplaced. The model uses a 2-stage model architecture for image processing.

**Figure 1:**
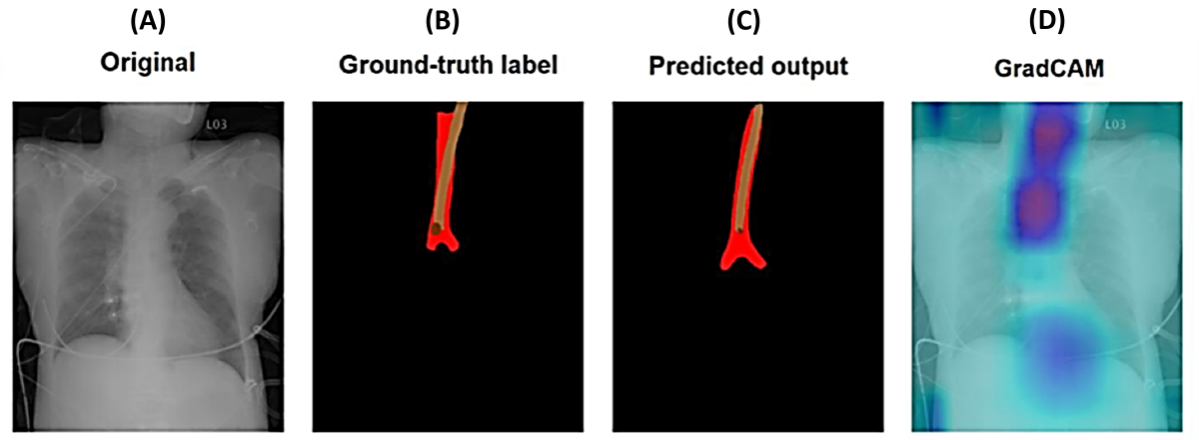
Summary of the model of localization and detection of malpositioned endotracheal tube (ETT). (A) original chest x-ray images. (B) Pixel-level annotations used by the labelers, including the ETT (brown) and the trachea and main bronchi (red), with dark brown dots indicating the ETT tip. (C) Segmentation masks generated by the segmentation model. (D) The gradient-weighted class activation mapping (Grad-CAM) results, highlighting that the classification model primarily relied on the region surrounding the ETT tip for its inferences.

The model uses a 2-stage architecture: DeepLabv3+ with ResNeSt50 as the backbone for segmenting the ETT, trachea, and main bronchi, followed by DenseNet121 for classifying ETT misplacement. Transfer learning was applied, using pretrained ImageNet weights fine-tuned on the dataset. The system demonstrated excellent performance. For segmentation, the Dice coefficients were 0.854 (NTUH-20 dataset) and 0.839 (NTUH-YB dataset), accurately delineating ETT contours. Mean tip localization errors were 1.107 cm (95% CI 0.807-1.513; NTUH-20) and 1.118 cm (95% CI 0.956-1.319; NTUH-YB). For classification, the system achieved area under the curve (AUC) scores of 1.000 (NTUH-20) and 0.994 (NTUH-YB) for ETT presence, and 0.847 (NTUH-20) and 0.734 (NTUH-YB) for ETT misplacement. It excelled in detecting one-lung intubation, with AUC of 0.991 (NTUH-20) and 0.966 (NTUH-YB), highlighting its robustness in critical scenarios. Results are summarized in Figure 1.

#### The Model for Misplacement Detection of NGTs

A total of 7378 portable CXRs were collected from NTUH Taipei main hospital and Yunlin branch (2015 to 2020) and annotated for NGT localization and malposition. The CAD system used DeepLabv3+ (ResNeSt50 backbone) for segmentation and DenseNet121 for classification. Testing on NTUH-20, NTUH-YB, and CLiP datasets demonstrated strong performance.

The segmentation model achieved Dice coefficients of 0.665 (NTUH-20) and 0.646 (NTUH-YB), with mean NGT tip localization errors of 1.64 cm and 2.83 cm, respectively. The classification model demonstrated strong performance, with an AUC of 0.998 for NGT detection and 0.964 (NTUH-20) and 0.991 (NTUH-YB) for malposition detection.

In summary, this deep learning–based model for tube misplacement detection demonstrated excellent performance in detecting and localizing ETT and NGT. Its accuracy and reliability highlight its potential for broad applications in emergency and intensive care settings.

#### The Model for Pneumothorax Detection

A deep learning–based dual-model system was developed for diagnosing and localizing pneumothorax on portable supine CXRs in emergency and intensive care settings. Images from NTUH (Taipei main hospital and Yunlin branch), collected between 2015 and 2020, were divided into training (1571 images) and test (1071 images) datasets, with pixel-level annotations.

The detection system used EfficientNet-B2, DenseNet-121, and Inception-v3 for classification, and Deformable DETR, TOOD, and VFNet for localization. The segmentation system used UNet for both classification and localization. The detection system achieved an AUC of 0.940, and the segmentation system achieved an AUC of 0.979, both performing similarly to radiology reports. The detection system achieved a Dice coefficient of 0.758, and the segmentation system achieved a Dice coefficient of 0.681, showing high accuracy.

**Figure 2:**
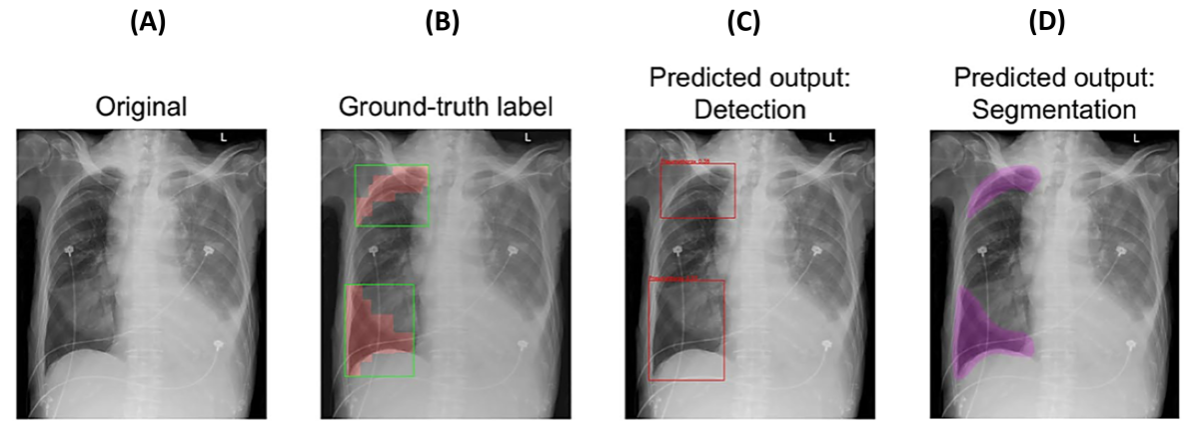
Summary of the model for pneumothorax detection. (A) Original image. (B) Boundary boxes (green rectangles) and segmentation masks (red regions) after preprocessing by the detection and segmentation model systems. (C) Boundary boxes (red rectangles) predicted by the detection model system. (D) Segmentation masks (purple regions) predicted by the segmentation model system.

These systems demonstrate strong performance and generalizability, offering potential to assist health care facilities in prioritizing critical x-rays and notifying clinicians for timely interventions, improving patient outcomes. Results are summarized in Figure 2.

The AI-based computer-aided diagnostic system automatically detects and assesses catheter placement and pneumothorax, providing excellent prejudgment capabilities for potentially misplaced catheter images. It reduces delays between obtaining CXR images and clinical interpretation, thus improving clinician efficiency. This, in turn, helps to reduce adverse reactions from catheter misplacement and subsequent management of pneumothorax, thereby improving patient prognosis and reducing complications.

This study focuses on the clinical validation and deployment of this system rather than on algorithm development or segmentation metrics. Although several foundational studies involving segmentation performance and metrics such as the Dice coefficient were conducted within the NTUH health care system, this trial aims to extend the findings and tools developed in those earlier studies to a broader range of health care settings. To enhance the generalizability and applicability of the research across institutions with varying levels and organizational structures, we have included 3 participating sites: a medical center (NTUH), and 2 regional hospitals (Fu Jen Catholic University Hospital [FJCUH] and Min-Sheng General Hospital [MSGH]).

## Methods

### Research Design

This study adopts a 2-phase design, beginning with a pilot study followed by a multicenter cluster randomized clinical trial. The pilot phase focuses on integrating a CXR CAD system into the hospital’s medical information systems, ensuring smooth functionality within the clinical workflow. The system will undergo testing and initial evaluations in the emergency intensive care and ICU at NTUH over a period of 6 to 12 months.

Building on the pilot phase, the multicenter clinical trial involves ICUs from NTUH, FJCUH, and MSGH. A total of 18 ICU units will participate, with NTUH contributing 14 units and the remaining hospitals contributing 2 units each. These units will be randomized into AI-assisted and control groups. Stratification is based on ventilator use, with randomization conducted within each stratum using uniform distribution. The AI-assisted group allows physicians to access real-time predictions from the CAD system during patient care. These predictions, generated within seconds, focus on identifying critical conditions, such as NGT misplacement, ETT misplacement, and pneumothorax. However, physicians in the control group will adhere to standard procedures without viewing AI-generated predictions, though the CAD system will still analyze images for internal validation. To minimize potential bias, both the outcome assessors and data analysts will remain blinded to group allocation throughout the data collection and statistical analysis phases.

The primary outcome is patient all-cause mortality rate: Assess whether the in-hospital mortality rate decreases after the intervention, reflecting the AI-assisted detection system’s ability to identify life-threatening conditions in a timely manner and facilitate rapid clinical intervention.

The secondary outcomes are as follows:

1. Cause-specific mortality rate: a physician panel, blinded to group allocation, will adjudicate the cause of death among study participants. The focus will be placed on respiratory-related mortality, which is most relevant to the conditions targeted by the AI modules.
2. Length of hospital stay: evaluate whether the use of the AI-assisted system shortens hospitalization duration, particularly by enabling earlier detection of tube misplacement and pneumothorax, leading to timely treatment.
3. Time to misplacement detection: determine whether the AI system reduces the time required to identify misplaced tubes or pneumothorax, thereby enhancing the timeliness of clinical interventions.
4. Time to reposition ETT and NGT, and time to place a chest drain for pneumothorax.
5. Direct medical costs (eg, x-ray examinations, intubation fees, and hospitalization costs) and indirect medical costs (eg, CAD system costs and labor costs).

### Determination of Sample Size

**Figure 3:**
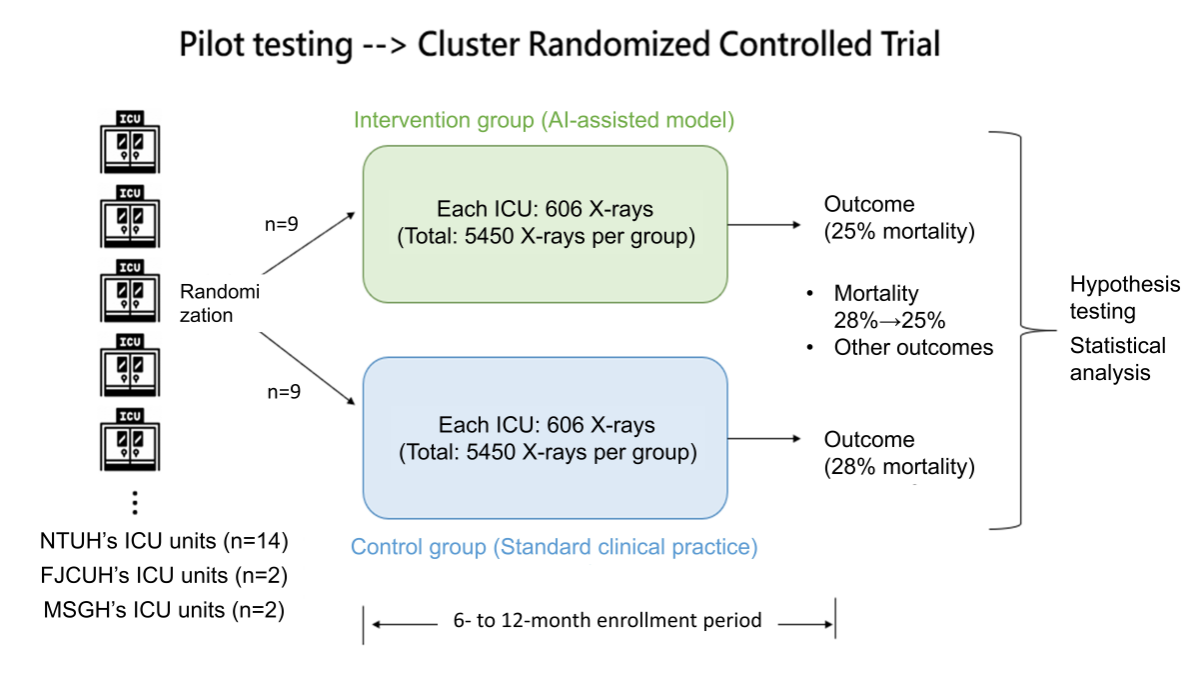
Illustration of study design. AI: artificial intelligence; FJCUH: Fu Jen Catholic University Hospital; ICU: intensive care unit; NTUH: National Taiwan University Hospital.

The primary outcome of the trial is to assess whether the CAD system can reduce in-hospital mortality. On the basis of data from the NTUH institutional database [19], the ICU mortality rate in 2023 to 2024 is estimated at 28%. A projected reduction to 25% was used for the sample size calculation. With an intraclass correlation coefficient of 0.01, a power of 80%, and a 1-sided α of 5%, each group requires 5450 patients, resulting in a total sample size of 10,900. This design is expected to provide sufficient power to detect clinically significant improvements in patient outcomes (Figure 3).

Because the causes of death in patients admitted in ICU may not necessarily be related to CXRs, adjustments to the primary outcome of the formal trial will be made following the pilot testing. This will involve evaluating and modifying the primary outcome (eg, time to detection of misplacement) to better align with the trial’s objectives and needs.

The conduct and reporting of the trial follow the CONSORT (Consolidated Standards of Reporting Trials) 2010 statement with the extension to cluster randomized trials [20].

### Recruitment and Enrollment

#### Phase 1: Pilot Study

During this phase, the emergency critical care (ECC) area and ICUs at NTUH will be selected as the pilot sites for system testing and preliminary evaluation. The implementation period will last approximately 6 months to 1 year from January 1 to December 31, 2025. Attending physicians, resident physicians, and advanced practice nurses from the ECC and ICUs will sign consent forms, enabling them to use the CAD system upon agreement.

Simultaneously, an exemption from patient informed consent will be requested, as the study is classified as minimal risk. The potential risks to study participants do not exceed those encountered by nonparticipants, and the exemption will not adversely affect their rights or welfare.

The inclusion and exclusion criteria are shown in Textbox 1.

**The inclusion and exclusion criteria for the pilot study.**

**Units**

- Inclusion criteria
- Emergency critical care or intensive care units at National Taiwan University Hospital
- The units included the patients requiring chest x-rays due to endotracheal intubation, nasogastric tube insertion, or ventilator use with a risk of pneumothorax.
- Exclusion criteria
- The unit supervisor does not agree to participate in the trial.
- The unit is unable to implement the artificial intelligence–assisted system (eg, no data connection or system support).

**Patients**

- Inclusion criteria
- Patients who are adults and require chest x-ray due to one of the following conditions: endotracheal intubation, nasogastric intubation, or the use of a ventilator with the potential to cause pneumothorax.
- Exclusion criteria for patients in isolation wards or pediatric wards
- Patients in isolation wards
- Patients in infant intensive care unit

The data collected includes the total number of patients in the ward, ventilator use, pneumothorax cases, CXR results, misplacement detection time, mortality rate, length of stay, ETT and NGT resetting times, pneumothorax drainage tube placement time, direct medical costs (eg, x-ray examinations, intubation fees, and hospitalization fees), and indirect costs (eg, CAD system and labor costs). System error messages will be recorded. Imaging data will not be collected, and the AI software will not undergo model training. A midterm evaluation at the end of phase 1 will determine the feasibility of phase 2.

#### Phase 2: Cluster Randomized Controlled Trial

Building on the results of phase 1, this phase will expand to include the ICUs of FJCUH and MSGH, conducting a cluster randomized controlled trial from January 1, 2026, to December 31, 2027, to evaluate the practical application of the CAD system in a multicenter setting, as well as to assess clinical and economic benefits. In addition, the attending physicians, residents, and specialized nurses in the ECC and ICUs will sign consent forms, which will allow them to use the CAD system. Patients will be exempted from providing informed consent, as the study poses minimal risk, and the potential risks for participants are no greater than those for nonparticipants. Exempting informed consent will not affect their rights. The inclusion and exclusion criteria are shown in Textbox 2.

The study will use cluster randomization, stratified by ventilator use frequency, to balance the risk of pneumothorax between the 2 groups and reduce potential confounding bias. Within each stratum, the ventilator use will be similar, while the difference in use between strata will be larger. A uniform distribution will be applied to assign each ward unit a random probability. On the basis of these probabilities, the units will be ranked and assigned to either the intervention or control group based on their odd or even rank.

In the control group, physicians will continue with the current diagnostic process and will not be able to view the model’s predictions. In the intervention group, physicians will be authorized to access the AI model’s predictions during patient care as an additional decision-making reference. These predictions will be generated in seconds and can help identify issues such as tube misplacement (eg, NGT and ETT) and pneumothorax through AI analysis of CXRs, which will alert the physician to review the images. Access to the model’s prediction interface was restricted to physicians in the intervention group, with access for the control group technically disabled through role-based permissions within the hospital information system. This measure ensured that physicians in the control group were not exposed to any AI-generated outputs. In addition, to promote consistent use of the CAD system within the intervention group, log-in records will be monitored, and reminders will be issued in cases of noncompliance.

Data collection included the total number of patients in the ward, the number of patients using ventilators, the number of pneumothorax cases, CXR results, misplacement detection time, mortality rate, length of stay, ETT resetting time, NGT resetting time, pneumothorax drainage tube placement time, direct medical costs (eg, x-ray examinations, intubation fees, and hospitalization fees), and indirect medical costs (eg, CAD system costs and labor costs). The implementation costs are expected to differ between the intervention and control arms. The intervention arm includes additional expenditures related to AI system deployment, such as system infrastructure, routine maintenance, training, and IT support. These costs are captured as part of the economic evaluation to assess whether the clinical benefits of AI-assisted care justify the associated investment. Imaging data will not be collected, and the AI software will not undergo model training.

**The inclusion and exclusion criteria for cluster randomized controlled trial.**

**Units**

- Inclusion criteria
- Emergency critical care or intensive care units at National Taiwan University Hospital, Fu Jen Catholic University Hospital, and Min-Sheng General Hospital.
- The units included the patients requiring chest x-rays due to endotracheal intubation, nasogastric tube insertion, or ventilator use with a risk of pneumothorax.
- Exclusion criteria
- The unit supervisor does not agree to participate in the trial.
- The unit is unable to implement the artificial intelligence–assisted system (eg, no data connection or system support).

**Patients**

- Inclusion criteria
- Patients who are adults and require chest x-ray due to one of the following conditions: endotracheal intubation, nasogastric intubation, or the use of a ventilator with the potential to cause pneumothorax.
- Exclusion criteria for patients in isolation wards or pediatric wards
- Patients in isolation wards
- Patients in infant intensive care unit

### AI Detection Tool

The CXR real-time CAD system is an AI-assisted diagnostic software in medical devices. It uses AI algorithms to analyze bedside CXR images and detect conditions such as malpositioned NGTs, ETTs, and pneumothorax. The system provides immediate feedback on detection results and notifies the physician to review the images for confirmation. If a tube misplacement is identified, the physician can promptly adjust its position; if pneumothorax is detected, the physician can provide appropriate treatment, such as chest tube drainage.

This system is classified as a medical device software under Taiwan’s Food and Drug Administration’s “Artificial Intelligent/Machine Learning-Based Software as a Medical Device, AI/ML-Based SaMD” regulations. It uses clinical data, such as images and biochemical markers, to autonomously learn and adjust its performance through AI programming. The outputs from the analysis are not intended to manage clinical treatments but provide immediate feedback to clinicians for timely interventions or treatments. The system’s data are sourced from patient examination files stored in the database and do not involve any invasive procedures.

### Data Integration Workflow

**Figure 4:**
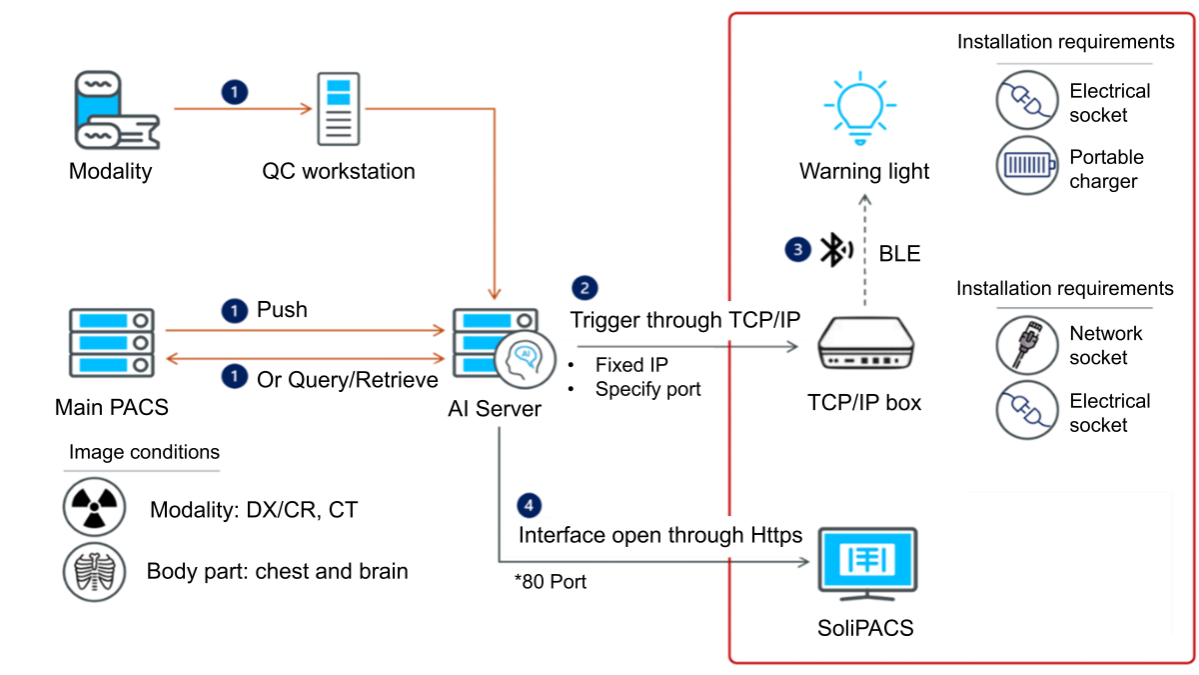
Data transmission flowchart for artificial intelligence (AI) server and medical information–system integration. BLE: Bluetooth low energy; CR: computed radiography; CT: computed tomography; DX: digital radiography; IP: internet protocol; PACS: picture archiving and communication system; TCP: transmission control protocol.

The workflow (Figure 4) ensures seamless data exchange between the AI server and the hospital’s medical information system, optimizing the use of AI in clinical decision-making. After CXR images are acquired, they are sent to the AI server via the QC workstation. To maintain data integrity, the system has a fail-safe mechanism: if transfer issues occur, the AI server queries or retrieves the images from the integrated information system or initiates a backup retrieval process directly. Once the AI server processes the images, critical findings are identified, and alerts are generated through the hospital’s internal messaging system, improving response times for urgent cases.

Health care professionals can access images and AI-generated annotations through SoliPACS by activating the “tool” feature. In addition, physicians can provide feedback on the AI’s diagnostic accuracy, facilitating model refinement. For research purposes, workflow configuration to enable AI access rights must be requested by ward physicians and specialist nurse practitioners in relevant departments.

### AI Computation Architecture

**Figure 5:**
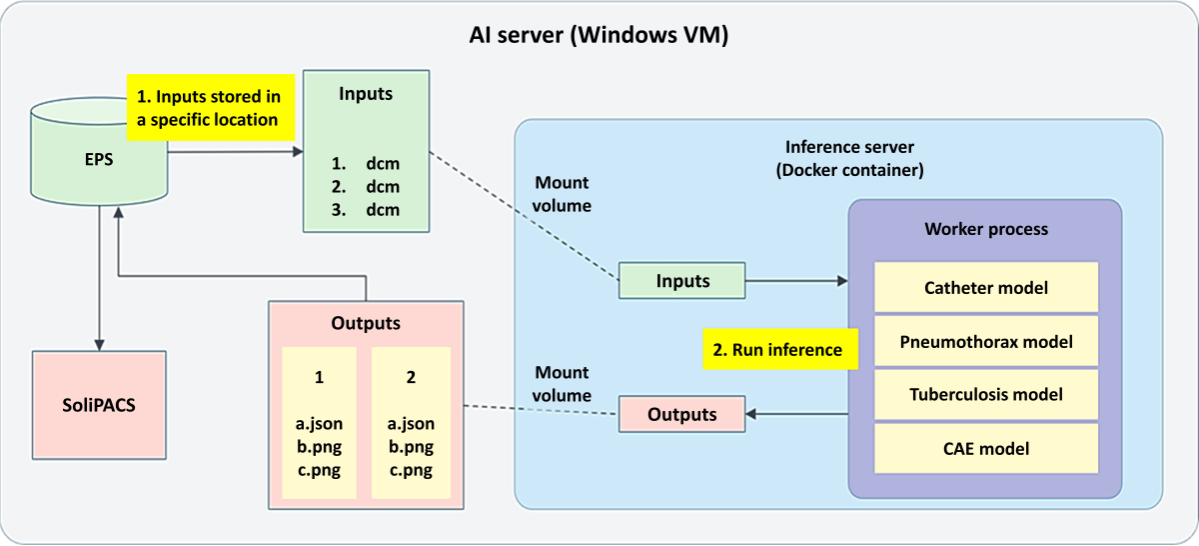
Artificial intelligence (AI) computing architecture diagram. EPS: EBM PACS Server.

The system uses a file exchange method to handle the transfer of DICOM files and output data (Figure 5). Batch processing capabilities allow users to process multiple images simultaneously by placing them in the “Inputs” folder. Built on an Inference Server architecture, the system loads the AI models into memory at container activation, eliminating the need to reload models for each operation. This architecture enhances computational efficiency, particularly for high-throughput image analysis. Critical alerts and findings are displayed in real time within the hospital’s information system, ensuring a streamlined workflow for health care providers.

### Statistical and Cost-Effectiveness Analysis

Statistical analysis will be conducted using SAS software (SAS Institute) to evaluate the outcomes of this study. Continuous variables, such as clinical outcomes and procedural times, will be summarized using descriptive statistics, including the number of observations, mean, median, SD, range, and 95% CIs. For categorical variables, frequency counts and percentages will be used to summarize the data.

In terms of clinical effectiveness, the primary outcome will be in-hospital mortality, and a proportional test will be used to compare the mortality rates between ICUs that have implemented the AI-assisted detection system and those that have not. The secondary clinical outcomes, which include the length of stay in the hospital, time to detect tube misplacements, and the time taken for medical interventions (such as repositioning of ETT and NGT and placement of pneumothorax drainage), will be analyzed using *t* tests for continuous variables. For categorical outcomes, including the rates of misplacement and other related interventions, chi-square test will be used. In addition, proportional tests will be conducted to examine the differences in the frequency of interventions across the 2 groups.

The study will also incorporate a health care technology and economic evaluation framework to assess the cost-effectiveness of the AI system alongside its clinical effectiveness. This evaluation will explore whether the clinical benefits derived from the AI system justify the associated resource consumption. Previous research by the team [21], such as the use of Markov models in gastric cancer screening and prevention, has demonstrated the utility of such economic evaluations, particularly in quantifying the cost-effectiveness of medical interventions and their impact on mortality reduction.

Cost-effectiveness analysis will be conducted using TreeAge software to compute the incremental cost-effectiveness ratio. This ratio will assess the additional cost required to achieve one unit of clinical benefit when using the AI system as compared to traditional methods. The incremental cost-effectiveness ratio will be calculated by dividing the cost of the intervention (which includes the medical, AI system, and maintenance costs) by the effectiveness, which will be measured through various clinical outcomes, such as reduced diagnostic time, decreased mortality rates, fewer misdiagnoses, and quality-adjusted life years (QALYs).

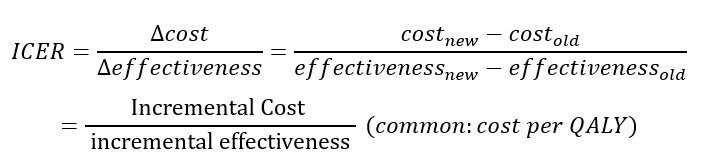



QALYs will serve as a composite measure of health outcomes by combining both the quantity and quality of life. The calculation of QALYs will involve multiplying the expected survival years after the intervention by a health state weight, which reflects the patient’s quality of life during those years. This weight ranges from 0 (death) to 1 (perfect health), and negative values may be used to indicate health statuses worse than death.

QALY = Years of Life × Health State Weight

To ensure the robustness of the findings, sensitivity analyses will be conducted, including both simple sensitivity analysis and probabilistic sensitivity analysis. These analyses will account for variability in model parameters and simulate different combinations of parameters to evaluate the stability of the cost-effectiveness conclusions. The results of these analyses will be assessed through acceptance curves and cost-effectiveness planes, which will allow a comparison of the cost-effectiveness of different treatment strategies under various assumptions.

### Ethical Considerations

The study was approved by the Research Ethics Committee of National Taiwan University Hospital (reference number: 202412055DINA), Fu Jen Catholic University Hospital (reference number: FJUH114448), and Min-Sheng General Hospital (reference number: CIRB2025001). The trial was registered on February, 2025, with ClinicalTrials.gov (NCT06842043). During data transmission and system access, it is crucial to ensure the full protection of patients’ medical information. To this end, physicians and advanced practice nurses from each ward involved in the study are required to request workflow permissions for AI system access. As AI systems typically rely on large volumes of medical imaging for training and optimization, any data breach or misuse could pose significant privacy risks.

The AI-assisted system may face potential challenges such as system malfunctions or delays, which could impact its reliability in real-time clinical decision-making. In critical care settings, detection delays or technical issues could result in treatment postponement. Furthermore, the AI system is integrated within the medical information infrastructure and must adhere to the institution’s information security management standards.

All data will be stored on dedicated, password-protected computers equipped with antivirus software. These research data and related information will be securely retained for a period of 5 years.

## Results

The implementation of the AI-assisted CXR CAD system is expected to yield substantial improvements in clinical outcomes and health care efficiency. By enabling timely identification of critical conditions such as misplaced ETT and NGT and pneumothorax, the system is anticipated to reduce in-hospital mortality rates from 28% to 25%. This improvement reflects the system’s potential to facilitate faster recognition of life-threatening situations and prompt clinical interventions.

Moreover, the use of the CAD system is projected to shorten the length of hospital stays, particularly by accelerating the detection of misplaced tubes and pneumothorax, which allows for timely correction and treatment. The time required for specific interventions, such as repositioning ETTs and NGTs or placing drainage tubes for pneumothorax, is also expected to decrease, further enhancing patient care efficiency.

From an economic perspective, the adoption of the CAD system is anticipated to reduce overall health care costs. This includes direct medical costs, such as imaging fees and hospitalization expenses, as well as indirect costs, such as personnel and system maintenance. By improving diagnostic accuracy and reducing delays in treatment, the system is expected to contribute to long-term cost savings while ensuring better health outcomes for patients.

## Discussion

### Principal Findings

The integration of the CXR CAD system is expected to significantly transform clinical workflows in emergency and critical care settings. By leveraging automated diagnostic capabilities, the study aims to address persistent challenges in timely and accurate identification of misplaced ETTs and NGTs and pneumothorax. The findings are anticipated to highlight the potential of CAD systems to improve patient outcomes, reduce health care costs, and provide a foundation for broader adoption of AI-based diagnostic tools in critical care environments. These results could position the CAD system as an essential component in advancing precision medicine.

### Limitations

Although this study is classified as low risk, the AI system used may present potential indirect risks. One concern is the possibility of false positives or negatives, where the system may inaccurately identify medical images, leading to missed diagnoses or misplacements, delaying treatment or causing unnecessary interventions.

Another risk is overreliance on the system by health care providers, particularly if they are unfamiliar with its use, which could affect decision-making accuracy. In addition, system errors or delays may occur, increasing clinicians’ workload if the AI system fails to provide timely assistance. In addition, as the system analyzes patients’ CXR images, there is a risk to patient privacy if proper data protection measures are not in place, potentially compromising confidentiality.

To address potential risks, a pilot study will validate the AI system’s sensitivity and specificity, ensuring reliability. The AI system serves only as a supplementary diagnostic tool, with final decisions made by physicians based on its outputs and clinical information. Health care providers will receive training on the system’s operation, limitations, and result interpretation to prevent overreliance. Regular feedback meetings will address issues as they arise. A monitoring protocol will log processing times and detect errors. In case of delays or malfunctions, standard diagnostic protocols will be followed. The AI software will also be approved by the NTUH Smart Medical Center to ensure data security and system integrity.

### Conclusions

Despite its limitations, the real-time CXR CAD system represents an innovative and promising tool for automatically identifying and detecting misplacement of ETTs and NGTs on CXRs while also assisting clinicians in diagnosing pneumothorax. This system has the potential to reduce detection time for tube misplacement and pneumothorax, lower patient mortality and hospitalization duration, and ultimately decrease health care costs. Following the evaluation protocol of this CAD system, its clinical accuracy and feasibility can be determined. If proven effective, further clinical trials may be considered to assess its broader impact.

## Abbreviations

AI: artificial intelligence
AUC: area under the curve
CAD: computer-aided detection
CONSORT: Consolidated Standards of Reporting Trials
CXR: chest x-ray
ECC: emergency critical care
ETT: endotracheal tube
FJCUH: Fu Jen Catholic University Hospital
ICU: intensive care unit
MSGH: Min-Sheng General Hospital
NGT: nasogastric tube
NTUH: National Taiwan University Hospital
NTUH-YB: National Taiwan University Hospital Yunlin Branch
QALY: quality-adjusted life year

## Data Availability

Data sharing is not applicable to this article as no datasets were generated or analyzed during this study. The development processes of the 3 models supporting this protocol are detailed in the primary supporting research articles [16-18]. Additional data can be obtained from the corresponding author upon reasonable request.
